# Right ventricular functions measured by cardiac magnetic resonance imaging in patients who underwent tricuspid valve surgery: implication for patients' outcome

**DOI:** 10.1186/1532-429X-17-S1-P176

**Published:** 2015-02-03

**Authors:** So Youn Shin, Joon-Won Kang, Won Jin Choi, Dong Hyun Yang, Tae-Hwan Lim

**Affiliations:** Department of Radiology, Asan Medical Center, Seoul, Korea (the Republic of

## Background

There are several evidences that right ventricular (RV) volume is important for the patients underwent surgery for tetralogy-of -Fallot, However, there is lack of evidence of RV volume after tricuspid valve surgery. Our aim was to evaluate right ventricular (RV) function using cardiac magnetic resonance imaging (CMRI) in patients who underwent tricuspid valve surgery for identifying predictors of poor postoperative prognosis.

## Methods

From 2008 to 2014, 842 patients underwent tricuspid valve operation at a single tertiary institution. All patients underwent preoperative echocardiography. Among them, 124 patients underwent preoperative CMRI to evaluate heart function including many parameters of volumetric measurement. Short-axis cine MRI images were analyzed using dedicated software. Ejection fraction, end-diastolic volume, end-systolic volume, and myocardial mass of both ventricles were evaluated. The primary composite endpoints or poor outcomes were defined as any cause of death, re-hospitalization due to aggravated heart failure, and redo-valve surgery. Using electronic medical record, baseline characteristics, past medical history, and patient outcome data were reviewed. We compared the CMRI and clinical variables between poor outcome and no poor outcome groups.

## Results

During follow-up (median of 417 days; range, 5-2232 days), 34 patients (30.6%) had at least one poor outcome, including 9 patients of all cause of death. In poor outcome group, left ventricular (LV) mass index and RV mass index were significantly greater (LV mass index, AUC 0.747, cut-off 61 g/m^2^; RV mass index, AUC 0.763, cut-off 27 g/m^2^). RV ejection fraction was significantly decreased and RV end-systolic volume index was enlarged (RV ejection fraction, AUC 0.684, cut-off 36%; RV end-systolic volume, AUC 0.700, cut-off 52ml/m^2^). Unadjusted Kaplan-Meier survival curves showed significantly lower survival rate in patients with large RV systolic volume and large ventricular mass index of both RV and LV.

## Conclusions

RV function measured by CMRI may provide prognostic information in patients who underwent tricuspid valve surgery. Measurement of both ventricular mass index and right ventricular end-systolic volume may help to identify patients with poor prognosis.

## Funding

N/A.

